# Immunolocalization of Extensin and Pectin Epitopes in *Liparis loeselii* Protocorm and Protocorm-like Bodies

**DOI:** 10.3390/cells13231985

**Published:** 2024-11-30

**Authors:** Michał D. Starke, Małgorzata Kapusta, Bartosz J. Płachno, Jerzy Bohdanowicz

**Affiliations:** 1 Laboratory of Plant Cytology and Embryology, Department of Plant Experimental Biology and Biotechnology, Faculty of Biology, University of Gdańsk, 59 Wita Stwosza St., 80-308 Gdansk, Poland; michal.starke@ug.edu.pl (M.D.S.); jerzy.bohdanowicz@ug.edu.pl (J.B.); 2 Bioimaging Laboratory, Faculty of Biology, University of Gdańsk, 59 Wita Stwosza St., 80-308 Gdansk, Poland; 3 Department of Plant Cytology and Embryology, Institute of Botany, Faculty of Biology, Jagiellonian University in Kraków, 9 Gronostajowa St., 30-387 Cracow, Poland; bartosz.plachno@uj.edu.pl

**Keywords:** cell wall, cell wall remodelling, endangered species, extensins, Orchidaceae, orchid germination, protocorms, protocorm-like bodies

## Abstract

*Liparis loeselii* (L.) Rich, an endangered member of the Orchidaceae family, is found in alkaline fens. With the declining populations of *L. loeselii*, there is a pressing need to reintroduce this species in Central Europe. As in vitro germination is a crucial tool for obtaining plants for introduction into the environment, we looked at the morphological changes occurring during the early stages of *L. loeselii* development in vitro. As the early stages of orchid development, especially the protocorm stage, are thought to be responsible for SAM formation and the initiation of symbiotic association, we focused on cell wall elements whose epitopes have been found in similar processes in other species: the extensin and pectin rhamnogalacturonan I (RG-I) side chain epitopes. We addressed the following questions: Does the cell wall of *L. loeselii* change its composition during the early stages of development, as noted in other species? Are there noticeable similarities in the cell wall to organs of different species whose function is to contact microorganisms? Are there regularities that allow the recognition of individual structures on this basis? Immunolocalization revealed changes in the distribution of certain extensins (JIM11 and JIM20) and RG-I (LM5 and LM6) side chain epitopes. Extensins, a type of cell wall protein, were observed during the initial stages of the formation of PLB and the shoot apical meristem of protocorms and PLBs. RG-I, on the other hand, was found to play a significant role in the development of the protocorm and PLB. In pseudobulbs, which appeared on the protocorms, extensins occurred in their storage part. However, RG-I side chains (1→4)-β-galactans (LM5), and (1→5)-α-L-arabinans (LM6) were not found in pseudobulbs. We revealed that a common feature of protocorms and PLBs was an increased amount of extensins, which were detected with the JIM11 antibody, and pectins, which were detected with the LM5 antibody, that were present together, which may prove helpful in determining the identity of the induced structures and distinguishing them from pseudobulbs. Thus, our study unveiled the role of extensins and RG-I during the growth of protocorms and PLBs. We suggest that PLBs may mimic the wall remodelling that occurs in protocorms, which indicates that using cell wall components is an invitation to be colonised by a fungal partner. However, this needs to be tested in future research. The findings of this research can help interpret future studies on the propagation, acclimatisation, and introduction of *L. loeselii* into the natural environment.

## 1. Introduction

**Figure 1:**
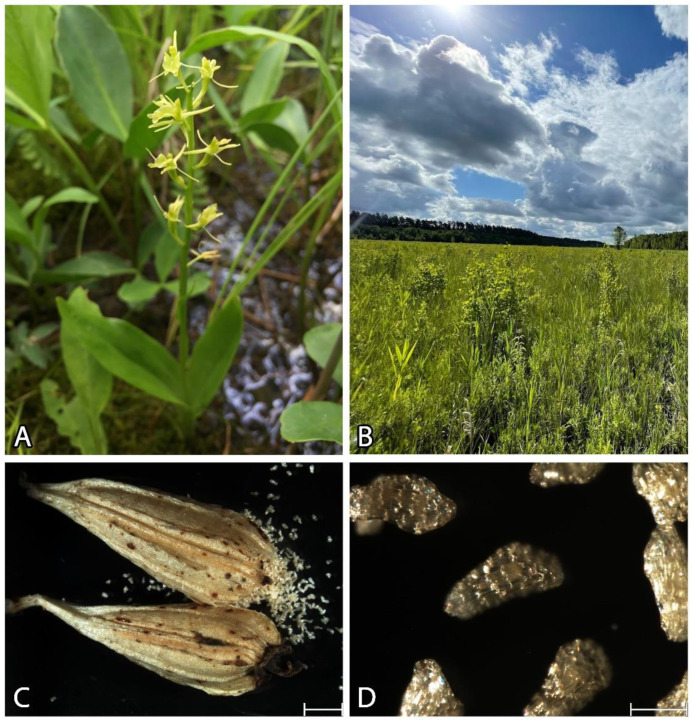
(**A**) *Liparis loeselii* in situ during flowering. (**B**) A natural occurrence site of *L. loeselii*, alkaline fens on the Rospuda River in Podlachia Voivodeship (Poland). (**C**) Mature capsules with seeds of *L. loeselii* were collected in situ. Scale bar = 2 mm. (**D**) Seeds of *L. loeselii*. Scale bar = 200 µm. (**A**,**B**) The images were obtained using an iPhone. (**B**) An ultra-wide angle lens was used. (**C**,**D**) The images were obtained by stereoscopic microscopy.

*Liparis loeselii* (L.) Rich. is a lithophyte, calciphilous, and light-demanding fen orchid (Figure 1) that is distributed in Europe and North America [1,2,3]. The occurrence of this amphiatlantic species is restricted to the temperate zone and mountainous regions southwards [2]. In Europe, the species has significantly decreased and is endangered because of land reclamation (e.g., land drainage), the intensification of land use, and the succession processes in abandoned meadows. In Poland, this species is considered vulnerable (VU) and has been placed under strict protection [2], which has resulted in a considerable decrease in its habitats [4,5].

Since orchid seeds do not have the structures that present in seeds of other plants, such as an endosperm and shoot apical meristem (SAM), and the number of cells that form the embryo is lower, the orchid embryo develops into a protocorm upon seed germination before forming a plantlet. The primary function of the protocorms is to create a SAM that allows the plant to continue to grow. This unique structure also establishes a symbiotic association with a compatible fungus. Although the formation of the structures that are typical of embryonic development occur in protocorms, the protocorm stage should not be considered to be an extension of embryonic development [6]. In the further development of the plant, the remaining structures are formed. In the case of *L. loeselii,* the SAM of protocorms forms a pseudobulb that then swells into a club-shaped mass [7].

Although the germination of most orchid species relies on symbiotic mycorrhiza, most of them can germinate asymbiotically when there are special medium and culture conditions [3,8]. To date, asymbiotic germination methods have been developed for many species in the Orchidaceae family [3,8,9,10,11]. In addition, in vitro cultures enable the induction of somatic embryogenesis and organogenesis, which makes it possible to increase the number of progeny plants that are obtained. These processes form new shoots, roots, calluses, or the somatic embryo. To date, these cultures have been used for many plant species, for both commercial and conservation purposes [10,12,13,14,15]. Moreover, unlike other species, protocorm-like bodies (PLBs) are observed in orchids; these structures resemble the protocorms that are formed on tissue explants and calluses in vitro [16]. Although the formation of PLBs resembles somatic embryos [17], molecular analyses indicate that the *SHOOT MERISTEMLESS* and *KNOTTED-LIKE HOMEOBOX* genes play a significant role during their formation, thus indicating that the PLB that are formed are a manifestation of organogenesis [18].

Tissue and organ morphology changes during the plant growth and developmental processes in plants are associated with the cell wall composition. It changes dynamically through structural modifications, the reorganisation of the cell wall components, and the synthesis and insertion of new elements into the existing walls. In addition, the cell wall participates in cell adhesion and intercellular communication and determines the shape of the cells [19]. The most abundant cell wall components are cellulose, hemicelluloses, pectin polysaccharides, and structural proteins. The important classes of cell wall proteins are glycine-rich proteins and hydroxyproline-rich glycoproteins (HRGPs), which include the arabinogalactan proteins (AGPs) and proline-rich proteins (PRPs) [20]. Observations of the modifications of the cell wall composition help us to understand the mechanisms of many processes, such as zygotic embryogenesis [21], somatic embryogenesis [20,21,22,23,24,25,26] organogenesis [17,27], pollen tube growth and ovule fertility [28,29,30,31,32], the response to environmental stress [33,34], and contact with a symbiont [35] or pathogen [36,37,38].

The family Orchidaceae includes about 28,000 species, which are divided into 736 genera [39]. This family abounds in many endangered species with a specific biology that is related to occupying specific habitats and reproduction. Many representatives of this family are also popular ornamental plants. Despite the abundance of species, the biology of representatives of the Orchidaceae is still poorly understood, especially when determining which cell wall components are involved in developing these plants [17,35].

The main goal of this study was to describe the morphology and analyse the distribution of cell wall components during the initial developmental stages of protocorms, seedlings, and PLBs of *L. loeselii*. Because the early stages of orchid development, especially the protocorm stage, are thought to be responsible for SAM formation and the initiation of a symbiotic connection, we focused on cell wall elements whose epitopes have been found in similar processes in other species. The first of the selected cell wall components is extensins, which have been found in other studies in differentiating cells or meristematic cells [17,40]. Epitopes of extensins were also detected in cells interacting with microorganisms [35,36,41]. The role of extensins is found in the involvement in cell wall construction and the growth and communication between the cell wall and the cytoplasm [42]. To localise the extensins, we chose antibodies JIM11 and JIM20, which detect different compositions of arabinose or galactose forming side chains of extensins [41]. The second immunolocalised component was the pectin rhamnogalacturonan I (RG-I) side chains, whose variable amount in the cell wall was observed during cell differentiation [19,26,43,44,45]. Changes in the distribution of these components were also observed during contact with the microorganism [46,47]. In this study, we selected two RG-I side chain epitopes: (1→4)-β-galactans detected with the LM5 antibody and (1→5)-α-L-arabinans detected with the LM6 antibody. RG-I side chain (1→4)-β-galactans are responsible for the cell wall rigidity and tip growth in some cells. However, (1→5)-α-L-arabinans are responsible for cell wall properties, such as the spatial buffering of homogalacturonan pectins, elasticity, resilience, expansion, porosity, water-holding capacity, and signalling [42,48]. In summary, the study aimed to answer whether, as in other species, the cell wall of *L*. *loeselii* changes its composition during the early stages of development? Are there noticeable similarities in the cell wall to organs of different species whose function is to contact microorganisms? Are there regularities that allow the recognition of individual structures on this basis?

## 2. Materials and Methods

### 2.1. In Vitro Culture

Mature seeds of *L. loeselii* (Figure 1) were collected from a fen on the Rospuda River in Szczebra, Podlachia voivodeship, Poland, in 2020, 2021, and 2022.

Before further processing, the seeds were placed in syringes, the end of which was closed with a nylon filter according to the method proposed by Ponert et al. [49] and incubated in 400 mg/L gibberellic acid (Duchefa Biochemie B.V., Haarlem, The Netherlands) overnight at 4 °C. Then, the gibberellic acid was removed, and the seeds were sterilised in the syringe for 30 s in 70% ethanol and 15 min in 1.5% calcium hypochlorite (Merck Life Science sp. z o.o., Poznań, Poland) containing 0.01% Tween-20 (Merck Life Science sp. z o.o., Poznań, Poland). After surface sterilisation, seeds were rinsed with sterile deionised water three times.

The seeds were placed in a semi-solid or liquid BM medium [3], and they were then cultured in the dark at 21 °C. All reagents used for in vitro cultures were purchased from Duchefa Biochemie B.V. (Haarlem, The Netherlands) and Merck Life Science sp. z o.o. (Poznań, Poland).

Photographs of the seeds and plants in vitro culture were taken under a Nikon SMZ 1500 stereoscopic microscope (Nikon Corporation, Tokyo, Japan) equipped with a digital DS-Fi1 camera (Precoptic, Warsaw, Poland). Photographs of the fens and *L. loeselii* in situ were taken with an iPhone (8 mp, ƒ/2.2 or ultra-wide angle lens 12 mp, ƒ/2.4; Apple Inc., Cupertino, CA, USA). The Adobe Photoshop CS6 software version 13.0 (Adobe Inc., San Jose, CA, USA) was used to select and edit the representative image sets.

### 2.2. Histochemistry and Immunohistochemistry

The detailed procedure for observing the histological sections and conducting the immunochemical analysis was described in Płachno et al. [50]. The plant material was fixed overnight at 4° C in 8% (*w/v*) paraformaldehyde (PFA, Merck Life Science sp. z o.o., Poznań, Poland) with 0.25% (*v/v*) glutaraldehyde (GA, Merck Life Science sp. z o.o., Poznań, Poland) in a PIPES buffer. The PIPES buffer contained 50 mM PIPES (piperazine-N, N′-bis [2-ethanesulfonic acid], Merck Life Science sp. z o.o., Poznań, Poland), 10 mM EGTA (ethylene glycol-bis [β-aminoethyl ether] N, N, N′, N′-tetraacetic acid, Merck Life Science sp. z o.o., Poznań, Poland), 1 mM MgCl2 (Merck Life Science sp. z o.o., Poznań, Poland), and 0.5% (*v/v*) Dimethyl sulfoxide (DMSO, Duchefa Biochemie B.V, Haarlem, The Netherlands), pH 6.8. It was then embedded in Steadman’s Wax and sectioned into 6 μm sections. The rehydrated sections were blocked with 1% bovine serum albumin (BSA, Merck Life Science sp. z o.o., Poznań, Poland) in a PBS buffer (137 mM NaCl, 2.7 mM KCl, 10 mM Na_2_HPO_4_ and 1.8 mM KH_2_PO_4_, Merck Life Science sp. z o.o., Poznań, Poland) and incubated with the primary antibodies JIM11, JIM20, LM5, and LM6 (Table 1) overnight at 4 °C. All primary antibodies were purchased from Plant Probes, Leeds, UK or Kerafast, Boston, MA, USA. The primary antibodies were used in a 1:20 dilution. The secondary antibody goat anti-rat conjugated with FITC was purchased from Abcam (Abcam, Waltham, MA, USA). Negative controls were created by omitting the primary antibody step, which caused no fluorescence signal in stained slides (Figure S1). The chromatin in the nuclei was stained with 7 µg/mL DAPI (Sigma Aldrich, Poznań, Poland) diluted in a PBS buffer. The samples were then cover-slipped using a Mowiol mounting medium, encompassing a mixture of Mowiol ^®^4-88 (Merck Life Science sp. z o.o., Poznań, Poland) and glycerol for fluorescence microscopy (Merck Life Science sp. z o.o., Poznań, Poland) with the addition of 2.5% DABCO (The Carl Roth GmbH + Co. KG, Karlsruhe, Germany). Immunofluorescence labelling was performed on at least several consecutive sections for each sample. Constant concentrations of reagents (antibodies, buffers, DAPI) were used for staining, and the same staining and washing times were used.

Selected slides presented for morpho-histological analysis were stained in a 0.01% solution of Fluorescent Brightener 28 (Calcofluor white; Merck Life Science sp. z o.o., Poznań, Poland) in PBS for 5 min to visualise cellulose in the cell walls. The chromatin in the nuclei was stained with 0,4 µg/mL propidium iodide (Merck Life Science sp. z o.o., Poznań, Poland) diluted in a PBS buffer.

The photos were viewed using a Nikon Eclipse E800 microscope (Nikon Corporation, Tokyo, Japan) equipped with a B-2A filter, a GFP custom filter, and a UV-2A, a DAPI filter combined with Nomarski contrast (DIC) with a digital DS-U1 camera (Precoptic, Warsaw, Poland), and a Leica DM6000B microscope (Leica Microsystems CMS GmbH, Mannheim, Germany) equipped with a GFP filter and DAP (DAPI) filter combined with Nomarski contrast (DIC) with a digital DFC450 C camera (Kawa.ska sp. z o.o., Zalesie Górne, Poland). The Leica Application Suite X software version 3.7.4. (LAS X, Leica Microsystems CMS GmbH, Mannheim, Germany) and Adobe Photoshop CS6 were used to select and edit the representative image sets.

### 2.3. Scanning Electron Microscopy

For the SEM, the material was fixed and then dehydrated and dried using supercritical CO_2_. The material was then sputter-coated with gold and examined at an accelerating voltage of 20 kV using a Hitachi S-4700 scanning electron microscope (Hitachi, Ltd., Tokyo, Japan), which is housed at the Institute of Geological Sciences, Jagiellonian University in Kraków, Poland [55]. The Adobe Photoshop CS6 was used to select and edit the representative image sets.

### 2.4. Statistical Analysis

All images used for measurements were taken using the same objective, with the same UV light intensity, the same time of exposition, and the same setting excitation and emission gain. The cell areas were measured by taking the average of ten randomly selected cells for each structure in three replicates (30 sizes per structure). They were measured manually using ImageJ software version 1.54d (NIH, Rockville, MD, USA).

The optical fluorescence intensity was calculated as the average of the pixel values from the green channel (fluorescence indicating the presence of the antibody) for 100 randomly selected areas on the walls in the fixed structures. Each selected structure type was measured in 3 replicates for 300 measurements per structure. The same method was used to measure the cell wall autofluorescence in the control variants, which was performed on the slides on which the labelling with primary antibody was omitted (Figure S2). Each measurement area was circular and was 2.121 µm^2^. They were measured manually using ImageJ software.

The data were analysed statistically using GraphPad Prism software version 1-0.3.1 (GraphPad Software, San Diego, CA, USA). An analysis of variance (one-way ANOVA) followed by post hoc Tukey’s honestly significant difference test was used to compare the mean values. For all of the analyses, the significance level was estimated at *p* < 0.05.

## 3. Results

### 3.1. Morpho-Histological Examination

**Figure 2:**
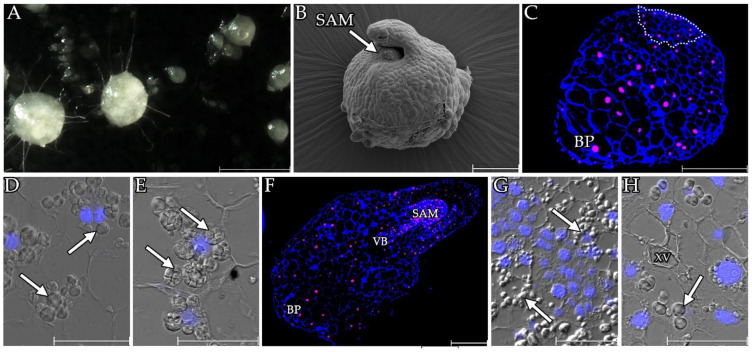
Germination of *Liparis loeselii* protocorms. (**A**) In vitro culture of protocorms 60 days after initiation. Scale bar = 1 mm. (**B**) Protocorms with a SAM (SAM) on the scanning electron microscope (SEM) photographs. Scale bar = 200 µm. (**C**) Young protocorm with poorly differentiated cells. Note that the cells within the place where the future SAM is developing (outline) are smaller than in other parts of the protocorm. Cells in the basal part (BP), on the other hand, are distinguished by their larger volume. Scale bar = 200 µm. (**D**) Formation site of the future SAM, notable starch grains (arrow). Scale bar = 50 µm. (**E**) The basal part of the protocorm with visible starch grains (arrow). Scale bar = 50 µm. (**F**) A developed protocorm with visible SAM (SAM). Scale bar = 200 µm. (**G**) Meristematic cells of a SAM and cells that were undergoing differentiation with visible starch grains (arrow). Scale bar = 50 µm. (**H**) Vascular bundle differentiation zone in the central part of the protocorm. Visible vessels of the xylem (XV). Visible starch grains (arrow) Scale bar = 50 µm. Images obtained using stereoscopic microscopy (**A**). Section stained with calcofluor (blue) and propidium iodide (red, (**C**,**F**)). Images were obtained by merging the fluorescence (blue, DAPI signals) with the DIC images (**D**,**E**,**G**,**H**).

After two months, most of the seeds (more than 30%) in the liquid and solid cultures had germinated and formed protocorms (Figure 2A). In the following weeks, there was a further enlargement of the protocorms as well as the formation of embryonic roots (rhizoids). After 60 days, most of the protocorms had a well-marked shoot apical meristem (Figure 2B).

**Figure 3:**
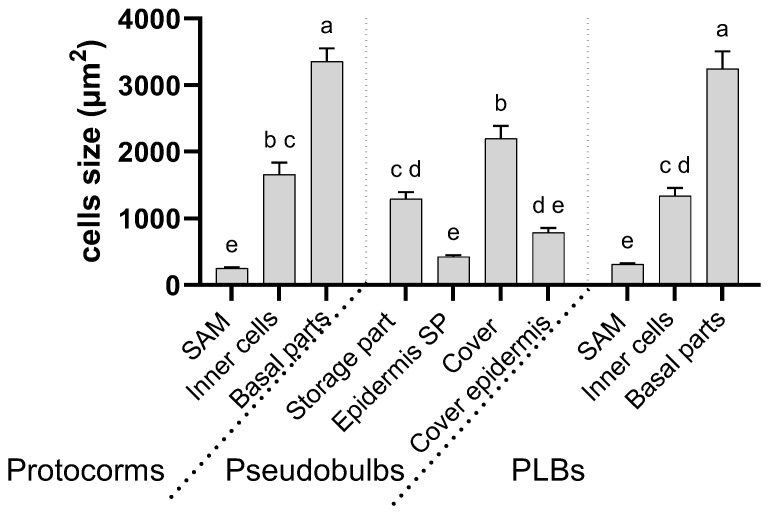
Comparison of the cell size in protocorms, pseudobulbs, and protocorm-like bodies (PLBs) of *Liparis loeselii* cultured in vitro. The cell size was calculated using ImageJ software. The values are the means ± SE, and different letters indicate significant differences (one-way ANOVA, *p* < 0.05) between the parts of the analysed structures.

Sections of the protocorms revealed significant amounts of starch grains in the cells (Figure 2C–H). Many starch grains were initially visible in the region responsible for forming the future SAM (Figure 2D). Only small single starch grains are visible in the meristematic cells of the SAM of the mature protocorms. In the zone of differentiating cells around the SAM, however, there was a noticeable deposition of starch as a storage material (Figure 2G). The protocorms were characterised by a gradient of cells with the cells in the basal part being significantly larger than those at the site of the SAM formation (Figure 2F–H and Figure 3). The difference in size was apparent even before the well-marked SAM appeared (Figure 2C–E). Also noticeable in the adult protocorms was the overlapping of the conductive bundles in the central part of the protocorm (Figure 2H).

**Figure 4:**
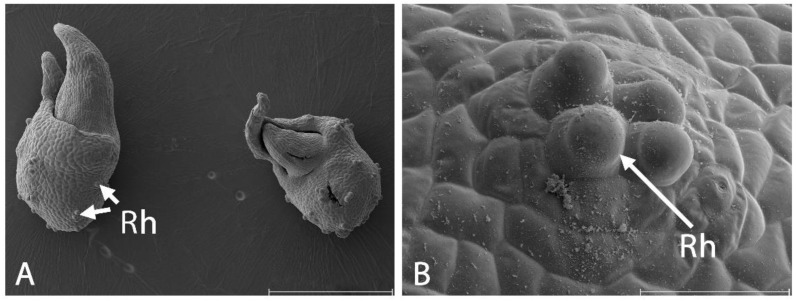
Pseudobulbs on *Liparis loeselii* protocorm from the liquid culture on the SEM photographs. (**A**) Protocorms with pseudobulbs from the liquid culture. Note the shortened rhizoids (Rh). Scale bar = 1 mm. (**B**). The shortened rhizoids (Rh) were outgrowths above the epidermis. Scale bar = 400 µm.

When the protocorms and pseudobulbs were grown in the liquid culture, the rhizoids only extended to a limited length, stopped growing, and formed raised cells above the epidermis (Figure 4).

**Figure 5:**
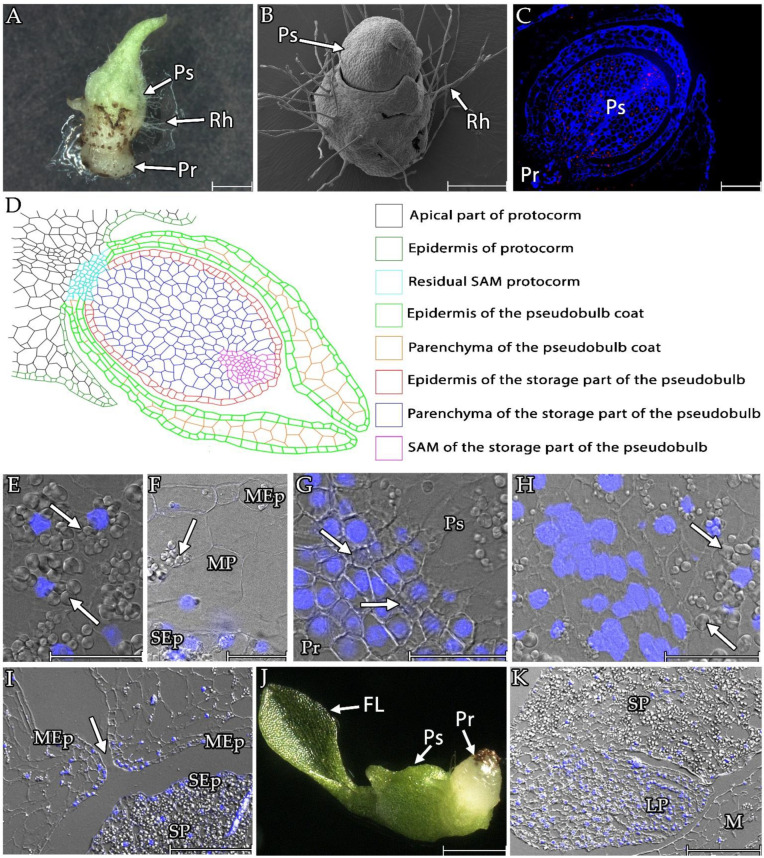
The appearance of pseudobulbs on the *Liparis loeselii* protocorms that were cultured in vitro. (**A**) A protocorm with pseudobulbs (Ps) after 120 days of the culture. Visible remains of protocorm (Pr) and numerous rhizoids (Rh). Scale bar = 1 mm. (**B**) Protocorms with pseudobulbs on the photographs (Ps) from the solid culture. Numerous visible rhizoids (Rh). Scale bar = 500 µm. (**C**) Pseudobulbs (Ps) on the protocorm (Pr). Scale bar = 200 µm. (**D**) Diagram illustrating the different tissues of the pseudobulb on the protocorm *L. loeselii*. (**E**) The parenchyma of the pseudobulb’s storage part contained cells with many starch grains (arrow). Scale bar = 50 µm. (**F**) Cells of the epidermis of the coat (MEp), parenchyma of the coat (MP), and epidermis of the storage part (SEp) had a low content of starch grains (arrow). Scale bar = 50 µm. (**G**) Junction site between the protocorm (Pr) and the pseudobulb (Ps). Visible vessel walls of the xylem of the vascular bundles (arrow). Scale bar = 50 µm. (**H**) Meristematic-like cells within the storage part of the pseudobulb. Starch grains were absent from the cells and appeared in the cells surrounding this zone (arrows). Scale bar = 50 µm. (**I**) The place above the top of the pseudobulb’s storage part (SP) where an aperture was created in the coat covering the pseudobulb (arrow). The connection between the inner and outer epidermis of the coat (MEp) was visible. Scale bar = 200 µm. (**J**) A seedling with the first leaf (FL) developed 200 days after germination. Visible remnants of the protocorm (Pr). Scale bar = 1 mm. (**K**) The leaf primordium (LP) grew from the base of the pseudobulb storage part (SP). The leaf appeared under the coat covering the pseudobulb (M). Scale bar = 200 µm. The images were obtained using stereoscopic microscopy (**A**,**J**). Section stained with calcofluor (blue) and propidium iodide (red; (**C**)). Images were obtained by merging the fluorescence (blue, DAPI signals) with the DIC images (**E**–**I**,**K**).

After about 90 days of the culture, a pseudobulb emerged at the top of the protocorm (Figure 5A,B). Microscopic sections revealed that the pseudobulb had a multi-layered structure (Figure 5C,D). The central part of the pseudobulb was a storage part that stored large amounts of starch in its cells (Figure 5D,E). The storage part was covered with a single layer of epidermis with cells that were smaller in volume to the covered part (Figure 3 and Figure 5D,F). At the point where the pseudobulbs connected with the protocorms, the presence of cells morphologically resembling those in the SAM of protocorms was noticeable (Figure 5D,G). The vascular bundles were also visible (Figure 5G). From where the protocorm connected to the top of the pseudobulb, a band of smaller cells resembling meristematic cells was noticeable (Figure 5H). In these cells, the amount of starch grains was negligible.

The storage part of the pseudobulb was covered by a multi-layered coat (Figure 5C,D). This coat comprised parenchyma that were bounded on two sides by a single-layer epidermis (Figure 5C,D,F). The mantle parenchyma cells were the largest within the pseudobulb (Figure 3) but contained significantly fewer starch grains than the storage part (Figure 5F,I). The mantle epidermis was also characterised by cells that were larger in volume than those that were found in the epidermis of the storage part (Figure 3). Like the mantle parenchyma, it only had a few starch grains (Figure 5F). The mantle in the upper part, furthest from the protocorm, remained unconnected. The epidermis covering the mantle’s inner and outer parts remained connected. As a result, the coat formed an aperture over the storage part of the pseudobulbs (Figure 5I).

After 160 days of cultivation, the first leaves began to appear (Figure 5J). Cross sections revealed that the leaves grew at the base of the pseudobulb and continued with the storage part (Figure 5K).

**Figure 6:**
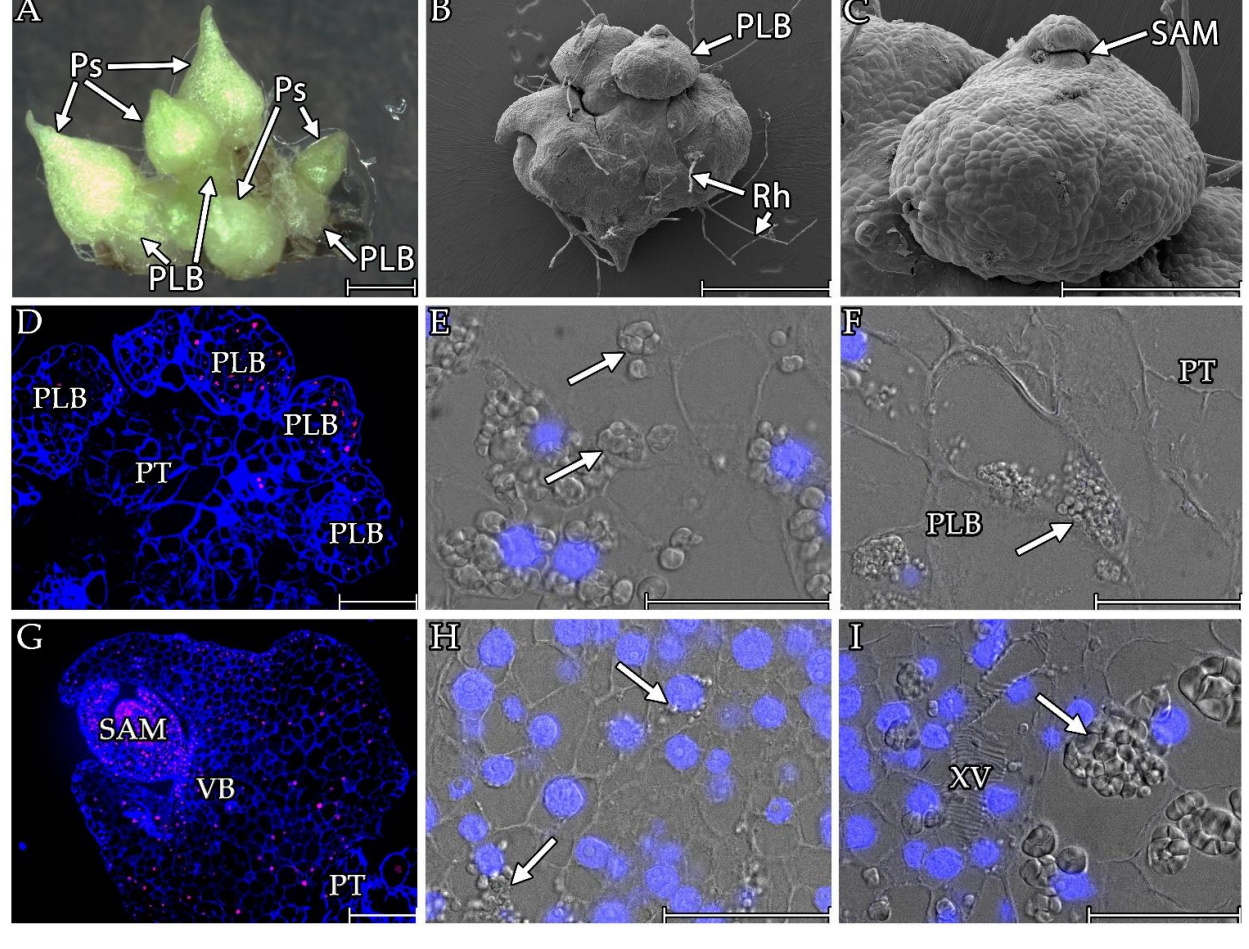
The appearance of the protocorm-like bodies and subsequent pseudobulbs on *Liparis loeselii* protocorms in the in vitro culture. (**A**) A protocorm with multiple pseudobulbs (Ps) and protocorm-like bodies (PLB). Scale bar = 1 mm. (**B**). Seedling with a PLB on the SEM photographs. Scale bar = 1 mm. (**C**) PLBs with a SAM on the SEM photographs. Scale bar = 400 µm. (**D**) Parental tissue (PT) on which young PLBs appeared. Scale bar = 200 µm. (**E**) The future SAM of the PLBs with a high content of starch granules (arrow) in the cells. Scale bar = 50 µm. (**F**) The boundary between the parental tissue (PT) and the PLBs. Visible starch grains (arrow) in the PLBs cells. Scale bar = 50 µm. (**G**) A PLB with differentiated cells and a visible SAM (SAM). In the central part, the formation of vascular bundles (VB) was noticeable. Visible connection to the parental tissue (PT). Scale bar = 200 µm. (**H**) The SAM of the PLBs with meristematic cells. Around the SAM, differentiating cells that deposited starch were visible (arrow). Scale bar = 50 µm. (**I**) The central part of PLBs had visible walls of xylem vessels (XV) and complex starch grains (arrows). Scale bar = 50 µm. The images were obtained using stereoscopic microscopy (**A**). The section stained with calcofluor (blue) and propidium iodide (red, (**D**,**G**)). Images were obtained by merging the fluorescence (blue, DAPI signals) with the DIC images (**E**,**F**,**H**,**I**).

Some protocorms (about 5%) formed protocorm-like bodies, which led to seedlings with multiple pseudobulbs (Figure 6). Morphologically, the nascent pseudobulbs (Figure 5A,B) strongly resembled the nascent PLBs (Figure 6A–C), although significant differences were observed in the histological sections. During the PLB formation, groups of small cells were observed above the mother tissue’s epidermal level to form the future PLB’s growth centre and then small spherical PLBs (Figure 6D). Like the protocorms, the young PLBs stored starch grains in their cells, including within the forming future SAM (Figure 6E). Starch granules were also visible in the PLB cells at the border with the parent tissue (Figure 6F). Like the protocorms, the developed PLBs were characterised by a significant cell size gradient between the different parts of the PLBs (Figure 6G). The meristematic cells that formed it were characterised by several individual starch grains. However, as in the case of the protocorms, starch deposition was noticeable in the cells that were undergoing differentiation in the zone around the SAM (Figure 6H). In the remaining parts, the starch grains were much more developed. The formation of vascular bundles was noticeable in the central part of the PLBs (Figure 6I).

### 3.2. Distribution of the Epitopes of JIM11 and JIM20 Antibodies

**Figure 7:**
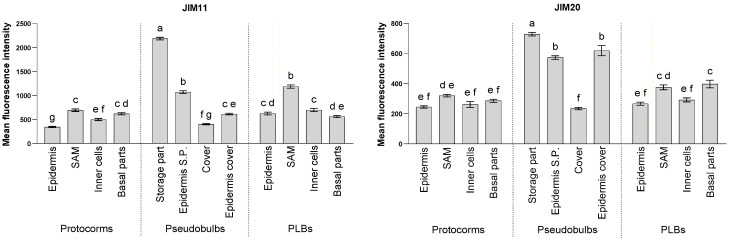
Comparison of the mean fluorescence intensity of the localisation of extensin in the cell wall in the analysed structures using the JIM11 and JIM20 antibodies. The fluorescence intensity was calculated using ImageJ software. The values are the means ± SE, and different letters indicate significant differences (one-way ANOVA, *p* < 0.05) between the parts of the analysed structures.

Antibodies JIM11 and JIM20, which recognise the different components chain of extensin, were selected to study the changes in extensin levels during the development of the protocorms, PLBs, and pseudobulbs of *L. loeselii*. JIM20 probably detects part or all of the structure containing the first three arabinoses and galactose of the extensin glycosyl fragment, while JIM11 probably detects the third and subsequent arabinose on the extensin glycosyl fragment. Extensins are consistently present at all stages of the protocorm, PLBs, and pseudobulbs development. However, the distribution of these molecules varies according to the structure and stage of development. There was also significantly stronger labelling with the JIM11 antibody compared to the JIM20 antibody in most of the structures that were examined (Figure 7).

**Figure 8:**
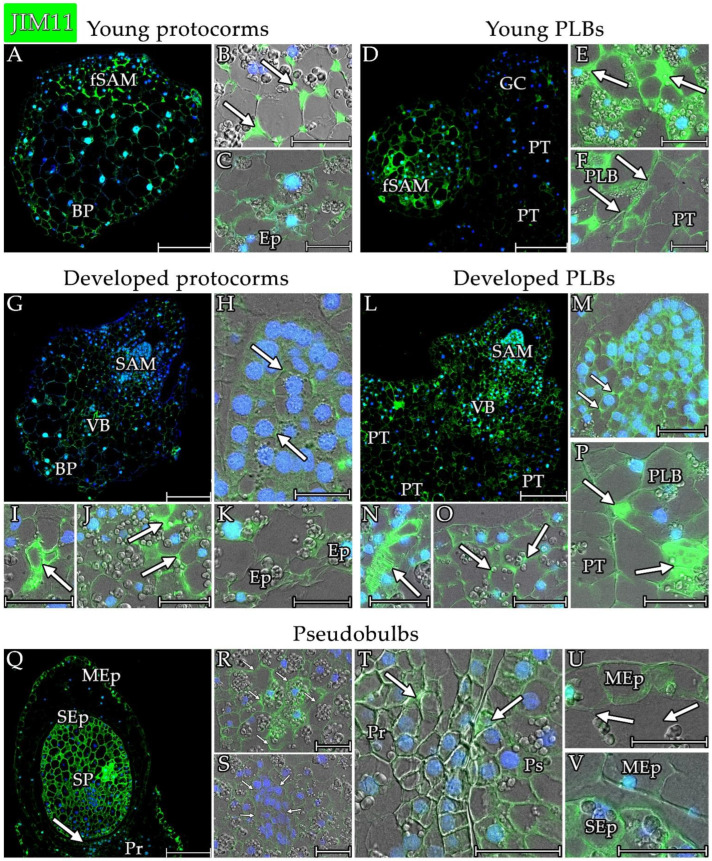
Distribution of the extensin epitope detected by the JIM11 antibody in the analysed structures of *Liparis loeselii*. (**A**) A young protocorm with poorly differentiated cells with the signal at the future SAM site (fSAM) and in the cell wall of the basal part (BP) cells. (**B**) Strong signal at the tri-cellular junctions (arrow) of the meristematic cells of the fSAM. (**C**) Detected extensin epitope in the cell wall of the cells of the basal parts and epidermis (Ep) of a young protocorm. (**D**) Young protocorm-like bodies that had separated from the parent tissue (PT) with a visible signal at the border crossing. Noticeable shaping of the future SAM site (fSAM). A strong signal was visible in the cells at the site of the formation of the next PLB with meristematic cells forming the growth centre (GC). (**E**) Strong signal in the extracellular matrix (arrow) within the meristematic cells of the future SAM. (**F**) The border between the PLB and parent tissue (PT) with a visible JIM11 epitope signal (arrow). (**G**) A protocorm with differentiated cells with a strong signal at the SAM site and in the middle zone where the vascular bundle (VB) differentiation occured. The cells in the basal part (BP) and many cells in the other parts of the protocorm also showed the JIM11 epitope signal. (**H**) Meristematic cells in the SAM of the protocorm with a signal at the cell wall and strong signal in tri-cellular junctions (arrow). (**I**) Vascular bundle differentiation site with a strong signal in the cell wall of xylem vessel (arrow). (**J**) The site below the SAM had a strong signal at the tri-cellular junctions and the extracellular matrix (arrow). (**K**) Basal parts of protocorm with signal in the cell wall. Signals were also seen in the epidermal walls (Ep). (**L**) PLB with differentiated cells and a visible SAM. The signal was visible in most of the cells of the structure. A strong extensin signal was visible within the SAM, within the site of vascular bundle formation (VB), and at the border between the PLB and the parent tissue (PT). (**M**) SAM meristematic cells had a signal in most of the cell walls and a strong signal in the extracellular matrix (arrow). (**N**) Vascular bundle differentiation site with a strong signal in the cell wall of xylem vessel (arrow). (**O**) The site below the SAM had a signal at the tri-cellular junctions and the extracellular matrix (arrow). (**P**) The border between the PLB and parent tissue (PT) with a visible signal in the extracellular matrix (arrow). (**Q**) Pseudobulbs had a strong JIM11 signal in all of the cells of the pseudobulb storage part (SP) and its epidermis (SEp). The signal was also visible in the outer epidermis of the coat covering the pseudobulb (MEp). The signal was also visible at the junction (arrow) of the pseudobulb with the protocorm (Pr). (**R**) A signal was visible in all of the cells of the pseudobulb’s storage part (arrow). (**S**) A signal was visible in the walls of meristematic cells of the storage part of the pseudobulb (arrow). (**T**) A signal was visible at the border between the pseudobulb (Ps) and protocorm (Pr). There was a strong signal at the site of conduction bundle formation and in the xylem vessels (arrow). (**U**) A signal was visible in the walls of the epidermal cells of the coat covering the pseudobulb (MEp). The signal was significantly weaker in the cell wall of the coat cells (arrow). (**V**) A strong signal was visible in the epidermal cell walls of the pseudobulb (SEp) storage part, and a weaker signal was present in the epidermal cell walls of the coat (MEp). Scale bar = (**A**,**D**,**G**,**L**,**Q**) 200 µm or (**B**,**C**,**E**,**F**,**H**–**K**,**M**–**P**,**R**–**V**) 50 µm. (**A**,**D**,**G**,**L**,**Q**) Images were obtained by merging the FITC (green) and DAPI signals (blue). (**B**,**C**,**E**,**F**,**H**–**K**,**M**–**P**,**R**–**V**) Images were obtained by merging the fluorescence (FITC and DAPI signals) with the DIC images.

The extensin epitope that was detected in the protocorms with the JIM11 antibody was detected in the cell wall of most of the cells (Figure 8A,G). During the initial stages of seed germination, when the shoot apical meristem (SAM) was not yet fully formed, extensins were found mainly in the cell walls of cells that would give rise to the meristem and basal region of the protocorm (Figure 7 and Figure 8A–C). Within the future SAM, a JIM11-associated fluorescence was observed, particularly at the three-cell junctions (Figure 8B). This signal also persisted in the SAM that formed in the protocorms, further noting a stronger signal in the intercellular spaces at the tri-cell junctions (Figure 8H). The signal of extensins that were found in JIM11, which formed an extracellular matrix around cells located in the periphery of the SAM, was also noticeable (Figure 8I). The extensin signal that was detected with the JIM11 antibody was also found within the site of conduction bundle differentiation. A powerful signal was found in the vessel walls of the xylem (Figure 8J). A strong signal was found in the cell wall of basal parts of protocorm (Figure 7 and Figure 8C,K). In the epidermis of the protocorms, the signal was weaker than in the inner parts (Figure 7 and Figure 8C,K).

In the PLBs (Figure 8D,L), the extensin signal that was detected by JIM11 was similar to the one that was localised in the protocorms (Figure 8A,G). However, the signal was significantly stronger than in the protocorms (Figure 7). During the early stages of PLB formation, the signal of the JIM11 epitopes on the cells at the boundary with the stem tissue was noticeable (Figure 8F). The signal in the extracellular matrix (Figure 8P) at the border between the PLBs and parental tissue also persisted at the later stages of the PLBs formation. At the early stages of PLB formation, a concentration of the JIM11 epitope was noticeable in the cells that were likely to form the future SAM (Figure 8E). The signal in these cells was localised in the walls of most of the cells of the future SAM, but it was also detected in the intercellular matrix (Figure 8E arrow). The signal also persisted in the protocorms whose SAMs were shaped. The signal came from the walls of most of the cells that formed the SAM (Figure 8M) and the intercellular matrix (Figure 8M arrows). The signal in the cell wall of the formed SAM was strongest in the rim of the PLBs (Figure 7). In the cells on the peripheral portions of the SAMs that had begun differentiating into other tissues, the signal of JIM11 epitopes was also noticeable, especially in intercellular matrices at the tri-cellular junctions (Figure 7 and Figure 8O). A strong signal was also localised within the cells that made up the conduction bundles, and a strong signal was detected in the xylem vessel wall (Figure 8N). JIM11 epitopes were also detected in the epidermal cell wall of the PLBs (Figure 8O), but the signal was significantly weaker than in the other parts of the PLBs (Figure 7).

In the cells of the pseudobulbs, the signal of extensins that were detected with JIM11 was the strongest of all the structures that were analysed (Figure 7 and Figure 8Q). The signal was strong in the central storage portion of the pseudobulb (Figure 7 and Figure 8R) in both the typical storage and meristem-like cells (Figure 8S). The signal was present evenly throughout the cell walls (Figure 8R,S). Additionally, the extensin signal that was detected by JIM11 inside some of the starch grain storage cells was noticeable (Figure 8R). The signal was detected in the epidermis of the storage part, but the signal was weaker than in the other cells of the storage part (Figure 7 and Figure 8V). The JIM11 antibody also detected extensins at the junction between the protocorm and pseudobulb, and stronger signals were detected in the vessel walls of the differentiated xylem (Figure 8T). The weakest signal was found in the walls of the cells of the pseudobulb coat (Figure 7 and Figure 8U arrows), except for the cells of the outer epidermis of the coat (Figure 7 and Figure 8U). The signal of the inner mantle epidermis was weaker (Figure 8V).

**Figure 9:**
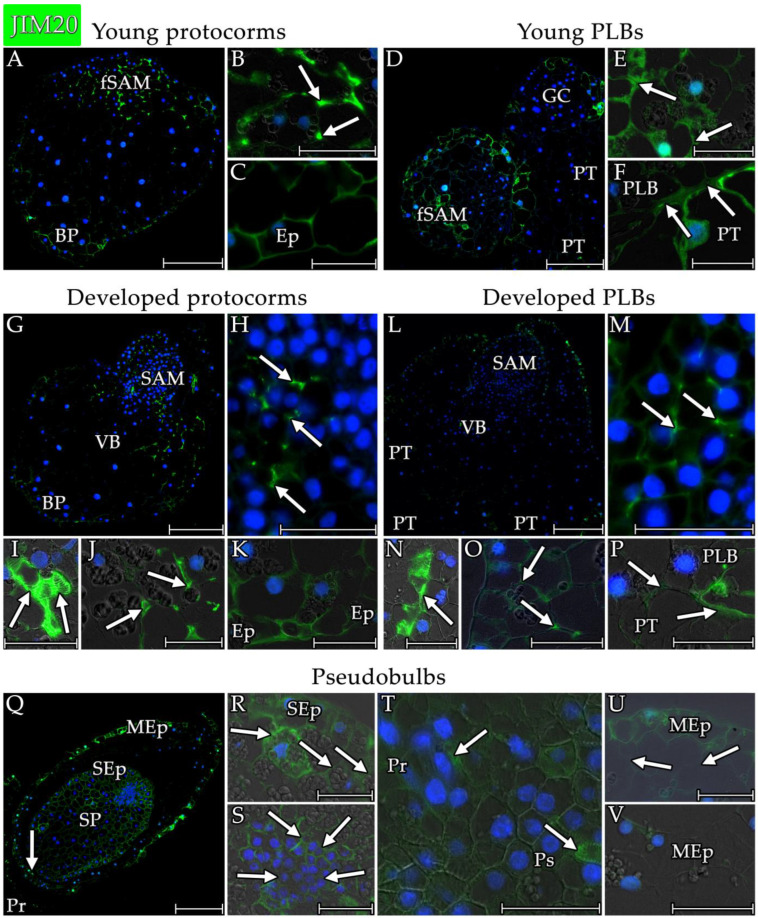
Distribution of the extensin epitope that was detected by the JIM20 antibody in the analysed structures of *Liparis loeselii*. (**A**) A young protocorm with poorly differentiated cells with the signal at the future SAM site (fSAM) and in the cell wall of the basal part (BP) cells. (**B**) Strong signal in the tri-cellular junctions (arrow) of the meristematic cells of the fSAM. (**C**) Detected extensin epitope in the cell wall of the cells of the basal parts and epidermis (Ep) of a young protocorm. (**D**) Young protocorm-like bodies separated from the parent tissue (PT) with a visible signal at the crossing border. Noticeable shaping of the future SAM site (fSAM). A strong signal was visible in the cells at the site of the formation of the next PLB with meristematic cells forming the growth centre (GC). (**E**) Strong signal in the extracellular matrix (arrow) within the meristematic cells of the future SAM. (**F**) The border between the PLB and parent tissue (PT) with a visible JIM20 epitope signal (arrow). (**G**) S protocorm with differentiated cells with a strong signal at the SAM site and in the middle zone where the vascular bundle (VB) differentiation occurred. Cells in the basal part (BP) and many cells in other parts of the protocorm also had a JIM20 epitope signal. (**H**) Meristematic cells in the SAM of a protocorm with a signal in the cell wall and a strong signal at the tri-cellular junctions (arrow). (**I**) Vascular bundle differentiation site with a strong signal in the cell wall of the xylem vessel (arrow). (**J**) The site below the SAM had a strong signal at the tri-cellular junctions and the extracellular matrix (arrow). (**K**) Basal parts of a protocorm with a signal in the cell wall. Signals were also visible in the epidermal walls (Ep). (**L**) A PLB with differentiated cells and a visible SAM. The signal was visible in most of the cells of the structure. A strong extensin signal was visible within the SAM, at the site of vascular bundle formation (VB), and at the border between the PLB and the parent tissue (PT). (**M**) The SAM meristematic cells had a signal in most of the cell walls and a strong signal in the extracellular matrix (arrow). (**N**) Vascular bundle differentiation site with strong signal in the cell wall of xylem vessel (arrow). (**O**) The site below the SAM had a signal at the tri-cellular junctions and the extracellular matrix (arrow). (**P**) The border between a PLB and the parent tissue (PT) with a visible signal in the extracellular matrix (arrow). (**Q**) The pseudobulbs had a strong JIM20 signal in all of the cells of the pseudobulb storage part (SP) and its epidermis (SEp). A signal was also visible in the outer epidermis of the coat covering the pseudobulb (MEp). A signal was also visible at the junction (arrow) of the pseudobulb with the protocorm (Pr). (**R**) There was a signal in all of the cells of the pseudobulb’s storage part (arrow). (**S**) A signal was visible in the walls of the meristematic cells of the storage part of a pseudobulb (arrow). (**T**) A signal was visible at the border between a pseudobulb (Ps) and a protocorm (Pr). A strong signal was visible at the site of conduction bundle formation and in the xylem vessels (arrow). (**U**) A signal visible in the walls of epidermal cells of the mantle covering the pseudobulb (MEp). The signal was significantly weaker in the cell wall of mantle cells (arrow). (**V**) A strong signal was visible in the epidermal cell walls of the storage part of the pseudobulb (SEp), and a weaker signal was present in the epidermal cell walls of the coat (MEp). Scale bar = (**A**,**D**,**G**,**L**,**Q**) 200 µm or (**B**,**C**,**E**,**F**,**H**–**K**,**M**–**P**,**R**–**V**) 50 µm. (**A**,**D**,**G**,**L**,**Q**) Images were obtained by merging the FITC (green) and DAPI signals (blue). (**B**,**C**,**E**,**F**,**H**–**K**,**M**–**P**,**R**–**V**) Images were obtained by merging the fluorescence (FITC and DAPI signals) with the DIC images.

In all of the structures that were analysed, the signal of extensins that was detected with the JIM20 (Figure 7 and Figure 9) antibody was significantly weaker than the signal with the JIM11 antibody (Figure 7 and Figure 8). In the protocorms (Figure 9A,G), the JIM20 antibody signal was mainly concentrated at the tri-cellular junctions and the extracellular matrix, particularly within the cells that were forming the future SAMs (Figure 9B) and the SAM (Figure 9G). This was also noticeable for other cells in the inner part of the protocorm (Figure 9J). In the basal part of the protocorms, the signal was more evenly distributed in the cell walls of the cells and also was detected in the epidermis (Figure 9C,K). The average signal in the epidermal cell walls of the protocorm was not significantly different from wall autofluorescence (Figure S2). A strong JIM20 antibody signal was also found within the xylem vessel wall (Figure 9I) at the point of the differentiation of the conductive bundles of the protocorm. The signal in the protocorm cells was uniform in the analysed structures of the protocorms (Figure 7).

In the PLBs (Figure 9D,L), the JIM20 epitope was found mainly in the SAM and the basal part where the PLBs fused with the parent tissue (Figure 7). This epitope was already detected in the cell wall during the formation of the future SAM (Figure 9D). In the formed SAM, a clear signal was localised in all of the meristem cell walls and stronger signals were found in the extracellular matrix at the junction of three cells (Figure 9M). A signal was also found near the SAM, particularly in the extracellular matrix at the three-cell wall junctions (Figure 9O). At the intersection between the PLB and parent tissue, the signal was present in the extracellular matrix (Figure 9F,P). A strong signal was also found in the xylem vessel wall within the conductive bundle formation zone of the protocorms (Figure 9N). As with the protocorms, the signal from the epidermis of PLBs was similar to the wall autofluorescence (Figure S1).

The JIM20 antibody signal was strongest among the structures that were analysed in the cell walls of the pseudobulbs (Figure 7 and Figure 9Q). The strong signal detected in the cells of the storage part in advance (Figure 9R,S), its epidermis (Figure 9V), and the epidermis of the coat covering the pseudobulb (Figure 7 and Figure 9U) were also significant. In the storage part, the distribution of the JIM20 epitope was uniform in the cell wall of all of the cells of both the cells with a large number of starch grains (Figure 9R) and the meristematic-like cells (Figure 9S). Extensins were also found in the cell walls at the junction of the pseudobulb and protocorm, particularly in the vessel walls of the xylem (Figure 9T). The weakest signal was found in the coat cells covering the pseudobulb (Figure 7 and Figure 9U arrow) and the inner epidermis of this structure (Figure 9V).

### 3.3. Distribution of the Epitopes of LM5 and LM6 Antibodies

**Figure 10:**
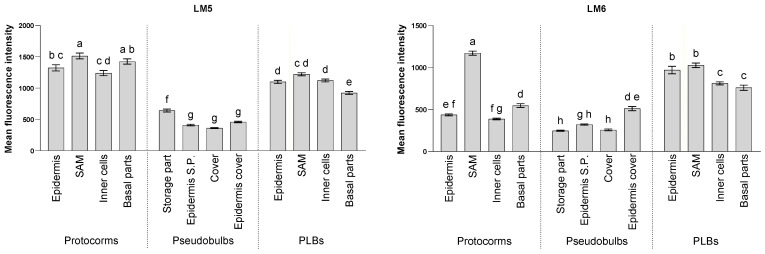
Comparison of the mean fluorescence intensity of pectin localisation in the cell wall in the analysed structures using the LM5 and LM6 antibodies. The fluorescence intensity was calculated using ImageJ software. The values are the means ± SE, and different letters indicate significant differences (one-way ANOVA, *p* < 0.05) between the parts of the analysed structures.

Pectins were localised using the LM5 and LM6 antibodies, which recognised the detected cell wall components in all of the analysed structures. However, their distribution changed significantly during the development of *L. loeselii* (Figure 10).

**Figure 11:**
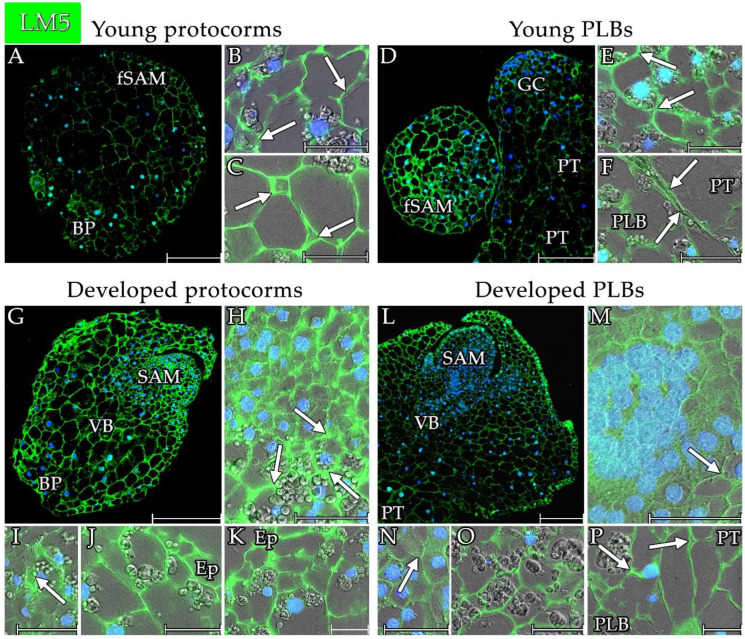
Distribution of the pectin epitopes that were detected by the LM5 antibody in the analysed structures of *Liparis loeselii*. (**A**) A young protocorm with poorly differentiated cells with signalling in most of its cell walls, especially in the future SAM (fSAM) and basal parts of the protocorm (BP). (**B**) Cells of the future SAM of the protocorm had signals in most of the cell walls and the extracellular matrix (arrows). (**C**) Cells of the basal parts of protocorm had signals in most of the cell walls and the extracellular matrix (arrows). (**D**) Young protocorm-like bodies with signalling in most of the cell walls, especially in the future SAM (fSAM). (**E**) Cells of the future SAM of the PLB had signals in most of the cell walls and the extracellular matrix (arrows). (**F**) The border between the parent tissue (PT) and a PLB with the signal at the border (arrows). (**G**) A developed protocorm with LM5 antibody signalling in most cell walls, including within the SAM, basal part (BP), and conduction bundle differentiation site (VB). (**H**) Signalling was present in the cell walls of the SAM meristematic cells. In the zone under the SAM, intense signals from the extracellular spaces at the differentiating cells (arrows). (**I**) Zone of the conduction bundle differentiation with signals in multiple walls including the xylem vessel walls (arrow). (**J**) LM5 antibody signals in the cell walls of the protocorm central mass. It also signalled in the cells of the protocorm epidermis (Ep). (**K**) The basal part of a protocorm with the LM5 epitope in most of the cell walls of cells, including epidermal cells (Ep). (**L**) Developed PLBs with signals in most of the cells in the SAM, the conduction bundle differentiation zone (VB), and the parental tissue (PT). (**M**) Signalling was present in cell walls of the SAM meristematic cells. In the zone under the SAM, intense signals from the extracellular spaces at the differentiating cells (arrows). (**N**) Zone of the conduction bundle differentiation with signals in multiple walls including the xylem vessel walls (arrow). (**O**) LM5 antibody signals in the cell walls of the central mass of the PLBs. (**P**) At the boundary between the parent tissue (PT) and the PLBs, a signal was visible in the entire cell wall. There was also a signal in the extracellular matrix (arrows). Scale bar = (**A**,**D**,**G**,**L**) 200 µm or (**B**,**C**,**E**,**F**,**H**–**K**,**M**–**P**) 50 µm. (**A**,**D**,**G**,**L**) Images were obtained by merging the FITC (green) and DAPI signals (blue). (**B**,**C**,**E**,**F**,**H**–**K**,**M**–**P**) Images were obtained by merging the fluorescence (FITC and DAPI signals) with the DIC images.

In the protocorms, the LM5 signal was present in most of the cell walls (Figure 10 and Figure 11A,G). The signal was mainly visible in the cell walls within the cells that were forming the future SAMs but was also noticeable in the extracellular matrix (Figure 11B). The signal was also present in the cell walls and extracellular spaces of the SAMs of the developed protocorms (Figure 11H). The signal from the SAM cell walls was strongest within the protocorms (Figure 10). A similarly strong signal was found in the basal part of the protocorms (Figure 10). This signal was localised mainly in cell walls but also in the extracellular matrix of cells (Figure 11C,K). A strong signal was also found at the site of vascular bundle formation, especially in the walls of the xylem vessels (Figure 11I). A strong signal was also present in the walls of the other cells forming the protocorms and in the epidermis of the protocorms (Figure 10 and Figure 11J).

The epitope of the LM5 antibody was localised less abundantly in the PLBs (Figure 11D,L) than in the protocorms (Figure 10). The strongest signal was found in the SAM cell walls (Figure 10 and Figure 11M). This signal was mainly found in the cell walls but was also found in the extracellular matrix (Figure 11M). The signal was also present during SAM formation in the younger protocorms (Figure 11E). The signal was also detected in the walls of the other cells forming the PLB (Figure 11O), including the cells at the junction of the PLB with the parent tissue (Figure 10 and Figure 11P). In the cells at the border between the PLBs and the mother tissue, LM5-localised pectins were also found in the extracellular matrix (Figure 11P arrow).

The LM5 antibody signal in pseudobulbs was much weaker than in the other analysed structures (Figure 10 and Figure S3). The strongest signal was detected in the cell wall of the cells of the storage part of the pseudobulbs. The LM5 antibody signal from the walls of the other parts of the pseudobulbs was about half as weak. The pectins that were detected with the LM5 antibody were also detected at the protocorms–pseudobulbs border and in the epidermis of the pseudobulb’s coat.

**Figure 12:**
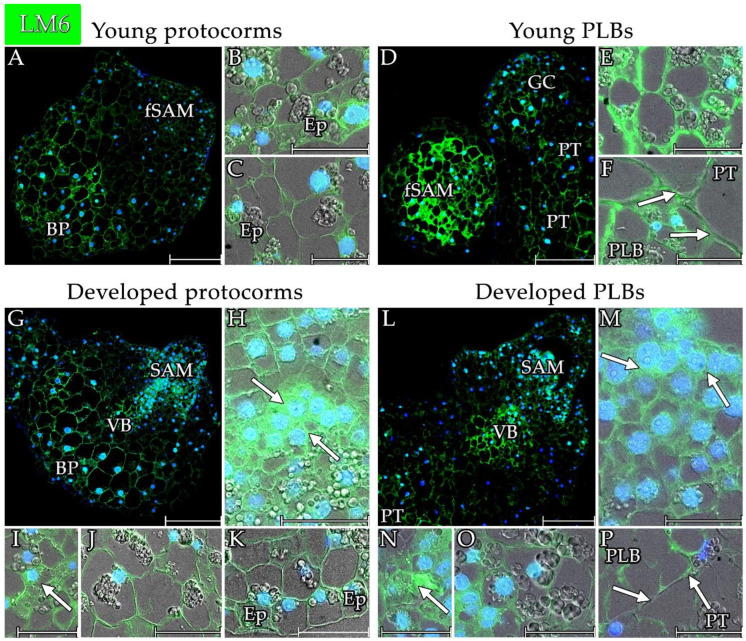
Distribution of the pectin epitope that was detected by the LM6 antibody in the analysed structures of *Liparis loeselii*. (**A**) A young protocorm with LM6 signalling was visible in the walls of most of the cells, particularly within the future SAM (fSAM) and basal part (BP). (**B**) The cells of future SAM had visible signalling within cell walls. The signal was also visible in the epidermis (Ep) above the SAM. (**C**) The basal part of the young protocorms had a signal in the cell walls. A signal was also present in the epidermis (Ep) at the basal part of the protocorms. (**D**) Young PLBs had LM6 signalling in most of their cells, particularly within the future SAM (fSAM) and the part that was connected to the parent tissue (PT). (**E**) The cells of future SAM had signalling in the cell wall. (**F**) The boundary between (arrows) a PLB and parent tissue (PT) with visible signals in the cell wall of cells of the PLB. (**G**) A developed protocorm with signals in most of its cells, particularly in the SAM, the vascular bundles (VB) differentiation site, and the basal part (BP). (**H**) A developed SAM of a protocorm with signals in the cell walls (arrows). (**I**) The site of the differentiation conducting bundles with signals in the cell walls of the cells including the xylem vessels (arrow). (**J**) The cells of the central part of a protocorm with signals in the cell wall. (**K**) The basal part of the developed protocorms had a signal mainly in the cell walls. A signal was also present in the epidermis (Ep) at the basal part of the protocorms. (**L**) A developed protocorm with signals in most of the cells, particularly in the SAM, at the vascular bundles (VB) differentiation site and the connection site with the parent tissue (PT). (**M**) A developed SAM of the PLBs with signals in the cell walls (arrows). (**N**) The site of differentiation was the conducting bundles with signals in the cell walls of the cells including the xylem vessels (arrow). (**O**) The cells of the central part of a PLB with signals in the cell wall. (**P**) The boundary between (arrows) a PLB and parent tissue (PT) with visible signals in the cell wall of the cells. Scale bar = (**A**,**D**,**G**,**L**) 200 µm or (**B**,**C**,**E**,**F**,**H**–**K**,**M**–**P**) 50 µm. (**A**,**D**,**G**,**L**) Images were obtained by merging the FITC (green) and DAPI signals (blue). (**B**,**C**,**E**,**F**,**H**–**K**,**M**–**P**) Images were obtained by merging fluorescence (FITC and DAPI signals) with the DIC images.

The LM6 epitope was detected throughout the protocorms of *L. loeselii* (Figure 10 and Figure 12A,G). The signal occurred within the formation of the future SAM in the cell walls (Figure 12B). In the SAM of the developed protocorms, the signal remained in the walls (Figure 12H). The LM6 signal in the SAM meristematic cell walls was strongest within the protocorms (Figure 10). In the basal part of the protocorms, the signal was in the cell wall of the cells (Figure 12C,K). The signal was also detected in the cell walls of the cells that comprised the central part of the protocorms (Figure 10 and Figure 12J) and at the differentiation site of the conductive bundles, including in the walls of the differentiating xylem bundles (Figure 12I).

Among the analysed structures, the strongest signal was detected in the PLBs (Figure 10 and Figure 12D,L). The signal was seen in the cell walls of the young PLBs. The signal was strong at the site of formation of the future SAM (Figure 12E) and, in part, where the PLBs connected to the parental tissue (Figure 12F). In the developed PLBs, the meristematic SAM cells were characterised by strong cell wall signals (Figure 10 and Figure 12M). A strong signal of the LM6 antibody was also observed within the site of the conduction bundle differentiation, particularly in the vessel walls of the xylem (Figure 12N). A signal was also visible in the walls of the cells that comprised the central part of the protocorms (Figure 10 and Figure 12O). As was the case in the young protocorms, at the junction between the PLB and parental tissue, the LM6 epitope within the cell walls was noticeable (Figure 12P).

The LM6 antibody signal within pseudobulbs was lower than for the other structures analysed (Figure 10 and Figure S4), including those within the storage section in the starch storage cells, in meristem-like cells, and the signals in the cell walls. This also characterised the cells of the storage part’s epidermis and the epidermis of the mantle. The pectins that were detected with the LM6 antibody were also detected at the protocorms–pseudobulbs border.

## 4. Discussion

### 4.1. Morphology

The germination of the *Liparis loeselii* seeds was similar to others that were observed in situ and reported in the literature [56]. Chlorophyl-free protocorms were observed to form from seeds on which pseudobulbs appeared, after which leaves grew. Subsequently, protocorms started to produce pseudobulbs. Protocorm-like bodies (PLBs) were also observed on which pseudobulbs appeared as they grew. It is possible that the formation of the PLBs, in the case of *L. loeselii*, is triggered by in vitro culture conditions or the rich medium that is used for protocorm culture. Teixeira da Silva et al. [57] showed that using a 3% sucrose-supplemented medium significantly increased the induction of PLBs from a shoot–tip hybrid *Cymbidium* compared to other polysaccharides.

The new pseudobulbs, which were obtained morphologically, resembled those that appeared in planta on *Malaxis paludosa* leaves, which Taylor [58] described as foliar embryos. As with those structures, a region of meristematic cells was observed, which was concentrated in the leaf stem tissue in *M. paludosa* and *L. loeselii* in the protocorms or PLBs. A mantle of giant cells in both species covered the main structure. Our observations indicate a consistent presence of meristematic cells spanning from the base to the apex of the primary structure, which were referred to by Taylor as the embryo. This band of cells differentiates the cells within the structure and increases in quantity towards the apex.

Like other higher plants, the Orchidaceae use starch as spare material, especially within their specialised organs such as tubers and rhizomes [59]. Starch is not stored in the cells of orchid seed embryos but appears in the form of starch grains during the development of the protocorms [60]. The appearance of starch grains in the cells of the *L. loeselii* protocorms was also evident (Figure 2). The deposition of starch grains also applied to the PLBs, as was previously described for leaf-induced PLBs of *Cattleya tigrina*, for example [61]. In the case of the PLBs of *L. loeselii*, we also noticed the deposition of starch grains that resembled those that were found in the protocorms (Figure 6). Significant amounts of starch grains were also noted in the cells of pseudobulbs, which emphasises the storage nature of these structures (Figure 5).

### 4.2. Changes in the Extensins Epitopes of the Explant Cells During the Culture

It is assumed that the most critical function of a protocorm is to form a functional SAM. Although the pathway of the SAM-forming cells is established during embryogenesis, significant remodelling is observed during the protocorm development [6]. In *L. loeselii*, while the cells of the future SAM were not significantly different from the other cells of the protocorm, an accumulation of the extensins that were detected with the JIM11 and JIM20 antibodies was noticeable in this area (Figure 8A and Figure 9A). Zhang et al. demonstrated the leading role of extensins in normal SAM development as well as during cell differentiation in *Nicotiana tabacum* [62]. Using a hydroxyproline synthesis inhibitor, 3,4-dehydro-L-proline (3,4-DHP), they showed that the inhibition of extensins in *N. tabacum* ovules and embryos in vitro leads to seedlings with an abnormally localised and formed SAM [40].

In *L. loeselii*, the JIM11 and JIM20 signals were concentrated in the extracellular matrix at the junction of three cells (Figure 8B and Figure 9B). This signal distribution was found in the SAM periphery during the protocorm’s further development. At the same time, the JIM11 and JIM20 signals in the SAM remained prominent in the cells in the central part of the SAM. JIM11 and JIM20 signals in the extracellular matrix were also found in late somatic embryos of banana *Musa* spp. AAA [40]. Zhang et al., as well as earlier studies by Tullio et al. of onion *Allium cepa* root development, showed that extensin inhibition has a relaxing effect on the cell wall in *N. tabacum*, which results in early cell elongation and a huge increase in cell size [40,63]. Therefore, it is probable that the presence of extensins in these zones limits the rapid release of cells from the SAM of protocorms in *L. loeselii*, thus enabling its proper functioning. It probably also enables controlled cell growth during differentiation. Tan et al. [64] suggest that extensins can interact with other wall components such as AGPs, and also with ions, to maintain cell membrane integrity during the wall-loosening processes as was noted; for example, in the ovular transmissive tissue of Asteraceae, which enabled the transition of the pollen tube [50].

Extensin epitopes, particularly the JIM11 epitope, were also detected in the basal part of the protocorm, which represents the compartment of the protocorm that is responsible for establishing a relationship with mycorrhizal fungus. The signal was detected in the cell wall (Figure 8C,K). Li et al. also detected the presence of the extensin JIM11 epitope in the basal part of the protocorm of *Dendrobium officinale*. They also noted that establishing a relationship with a symbiont increased the presence of this epitope in the cell walls and the interfacial matrix at the boundary between the cell membrane and the fungal wall [35].

The arrangement of the protocorms and PLB *L. loeselii* cells, especially for the early stages, resembled the one that was obtained by Lee et al. [17] for a *Phalaenopsis* ‘Taisuco Yellow’ cultivar. They indicated a crucial role of the extensins that were detected by JIM11 and JIM20 in the PLB formation. Similarly to the *P.* ‘Taisuco Yellow’ cultivar, in *L. loeselii*, we observed that tissues on which the PLB induction occurred were characterised by higher levels of extensins localised via JIM11 and lower, but also increased levels of extensins localised via JIM20.

Significant amounts of the JIM11 and JIM20 extensin epitopes were detected in the storage part of the pseudobulbs. The pseudobulb is a storage and perennating organ, so higher levels of the extensin epitopes could represent preparation for dormancy that is necessary for temperate plants. Wilmowicz et al. found that drought stress resulted in increased extensin levels in the root nodules and flower abscission zone in yellow lupin *Lupinus luteus* [65]. Additionally, regarding *Arabidopsis thaliana* seedlings, during salt stress, higher levels of the extensin epitope that is detected with the JIM11 antibody were found [66]. It is also possible that, as with protocorms, extensins are responsible for limiting the proliferation of the symbiont in the host cells [35]. Confirmation of this, however, requires further research on plants that are grown together with their symbiont.

Castilleux et al. reported a hypothesis that indicated differences between the epitopes that are detected by JIM11 and JIM20 antibodies. They noted that JIM20 recognises part or all of the structure containing the first three arabinoses and galactose from the extensin glycosyl fragment. JIM11, on the other hand, recognises the third arabinose and/or subsequent arabinose residues on the extensin glycosyl fragment [41]. The data obtained for *L. loeselii*, but also the data presented previously by other teams for *D. officinale* and *P.* ‘Taisuco Yellow’ cultivar, indicate that in the Orchidaceae family, the JIM11 extensin epitope is dominant over the JIM20 epitope during the early developmental stages [17,35]. The similarity in the number of epitopes that were detected, as well as in their composition, can also be seen in comparison with other monocotyledonous species, such as *Musa* spp. [40].

### 4.3. Changes in the Pectin Epitopes of the Explant Cells During the Culture

Pectin epitopes were detected, particularly within the cell walls of the protocorms and PLBs. The LM5 antibody signal that detects the pectin epitope (1→4)-β-D-galactan was present in the cell walls of the meristematic SAM cells. It was also strong in other cells, forming the protocorm and PLBs. Canaveze et al. made similar observations in the *Tabernaemontana catharinensis* apical shoot, detecting the signal in both meristematic and parenchyma cells [43]. In embryos in pea grains, the appearance of (1→4)-β-D-galactan in the cell walls occurred after cell expansion and the formation of the cell spaces but before dehydration and maturation. An increase in tissue firmness accompanied this [45]. Klassen et al. suggested that the galactan side chains of RG-I that interact with other cell wall components, such as xyloglucans and cellulose microfibrils, may regulate polymer separation and porosity in the apoplastic space. This mechanism probably protects the cell wall matrix from dehydration [67]. The importance of the pectin epitopes in water absorption is indicated by their presence in aerial roots of *Vanilia phaeantha* [68] and other epiphytic representatives of the family Orchidaceae [69]. Canaveze et al. point to the role of (1→4)-β-D-galactan in maintaining the firmness and adhesion of cells to each other [43].

The signal of the LM6 antibody that detects the epitope (1→5)-α-L-arabinan was detected in all of the protocorm walls and PLBs. However, a strong signal was observed mainly in the meristematic cell walls of the SAM. The LM6 epitope in *L. loeselii* was also detected in the cells that were acquiring meristematic potential, which is similar to the results that were obtained by Kuczak and Kurczyńska during the induction of somatic *Daucus carota* embryos [19]. Pérez-Pérez et al. detected an increase in the level of this epitope during the somatic embryogenesis of *Quercus suber* [26]. Canaveze et al. indicated a similar distribution of this epitope for the shoot apex of *T. catharinensis*. The signal was detected in the meristematic cell walls and was not found in the parenchyma cells. The arabinan chains that are present in the pectin component of the cell wall increase its fluidity, which may facilitate apoplastic transport. The presence on RG-I of side chains in the form of (1→5)-α-L-arabinan affects the elasticity of the cell wall, which may increase their strength during growth [43]. Pilarska et al. noted a similar sequence of RG-I side chain epitopes that appear during the formation of *Trifolium nigrescens* somatic embryos. They also pointed out the importance of the arabinan chain in loosening the cell wall [44].

In addition to their involvement in plant morphogenesis, pectins are also involved in microbial interactions [46,47]. Therefore, an increased presence of pectins in the protocorm cells may be due to their readiness to establish symbiosis with mycorrhizal fungi. The PLBs may then mimic the wall remodelling that occurs in the protocorms.

MacDougall et al. showed that extensins and pectins can form a gel that is able to stabilise the cell wall [70]. In the protocorms and PLBs, the signal of the pectic epitopes overlapped with the signal of the extensin epitopes. The mutual interaction of these compartments may also contribute to the formation of the cell walls, which is necessary for morphogenesis and the establishment of a connection with the mycorrhizal symbiont.

## 5. Conclusions

Our article is the first to show the changes in the cell wall composition during orchid germination and development so accurately. Our research shows that the distribution pattern of extensins changes during protocorm formation and the subsequent growth of *Liparis loeselii,* a peatland orchid of temperate climates. In addition, we show that during the orchid’s development from a protocorm to seedling, the cell walls are remodelled in terms of the specific pectins that are detected with the LM5 and LM6 antibodies. Moreover, we indicate that using the LM5 antibodies and JIM11 can help determine the identity of emerging PLB-type structures. PLBs may mimic the wall remodelling that occurs in the protocorms, thus showing that using cell wall components is an invitation to be colonised by a fungal partner. However, this hypothesis requires further research involving symbiotic fungi.

## Figures and Tables

**Table 1:** List of the primary rat monoclonal antibodies that were used in the presented study.

Antibody	Epitope	References
JIM11	extensin/HRGP glycoprotein	[51,52]
JIM20	extensin/HRGP glycoprotein	[51,52]
LM5	linear tetrasaccharide in (1→4)-β-D-galactans (RG I side chain)	[53]
LM6	linear pentasaccharide in (1→5)-α-L-arabinan (RG I side chain)	[54]

## Data Availability

Data are contained within the article.

## Supplementary Materials

The following supporting information can be downloaded at https://www.mdpi.com/article/10.3390/cells13231985/s1, Figure S1. Negative control for immunochemical analysis. Figure S2. Comparison of the mean fluorescence intensity of the FITC antibody conjugated to the JIM11, JIM20, LM5, and LM6 antibodies and cell wall autofluorescence (CTRL) in the cells of the analysed structures. Figure S3. Distribution of the pectin epitopes that were detected by the LM5 antibody in the analysed structures of *Liparis loeselii*—pseudobulbs. Figure S4. Distribution of the pectin epitope that was detected by the LM6 antibody in the analysed structures of *Liparis loeselii*—pseudobulbs.
