# Supplementary material for: Immunolocalization of Extensin and Pectin Epitopes in Liparis loeselii Protocorm and Protocorm-like Bodies
Source: Cells. 2024 Nov 30;13(23):1985. doi: 10.3390/cells13231985 (PMC11640368; doi:10.3390/cells13231985)
Supplement: Supplementary file 1 [file cells-13-01985-s001.zip › cells-3300158-supplementary.pdf]

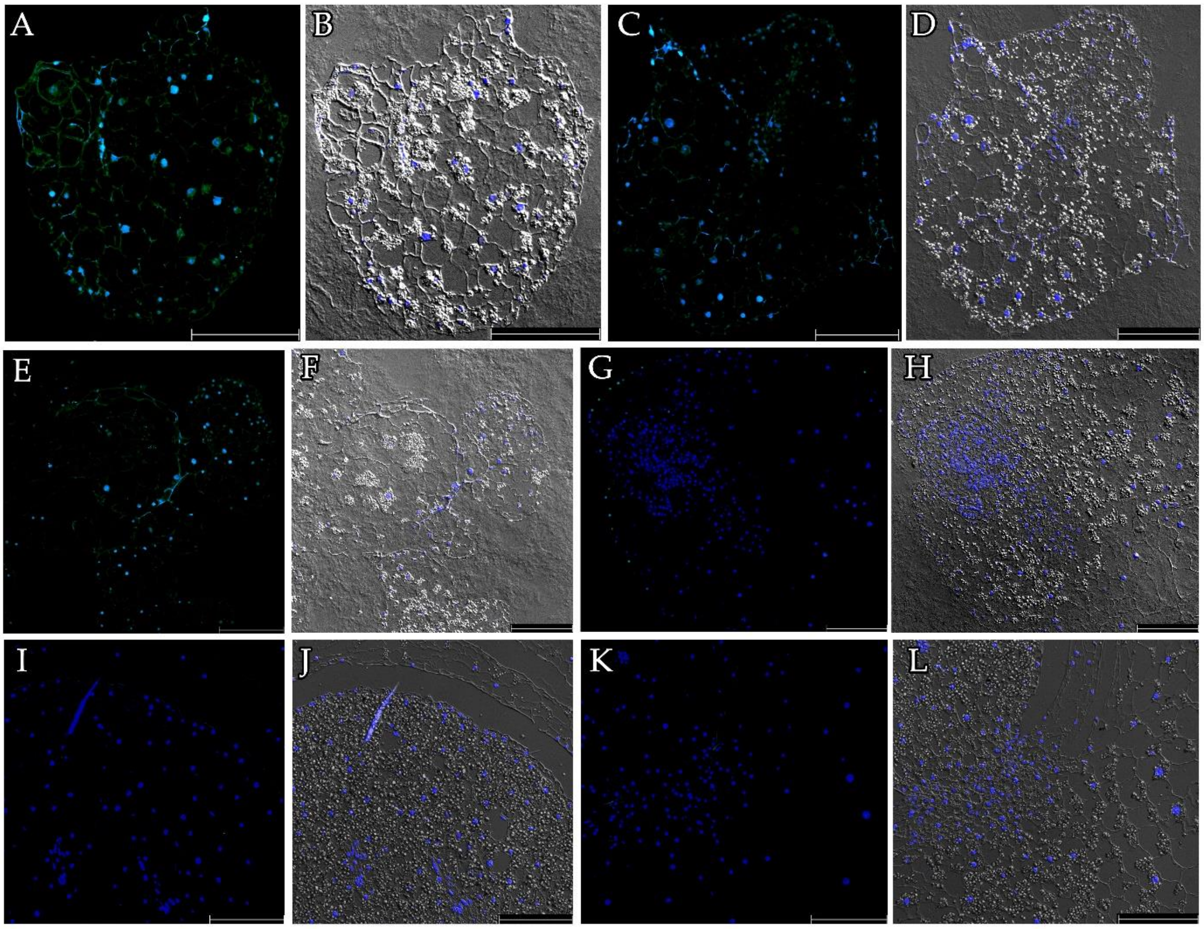

Figure S 1 Negative control. (**A** and **B**) A young protocorm with poorly differentiated cells. (**C** and **D**) A developed protocorm with a visible SAM. (**E** and **F**) A PLB with poorly differentiated cells diverging from the parental tissue. (**G** and **H**) A PLB with differentiated cells and a visible SAM. (**I**-**L**) Pseudobulbs on a protocorm. (**I** and **J**) The apical part of the pseudobulb. (**K** and **L**) The basal part of the pseudobulb. Scale bar = (all images) 200µm (**A, C, E, G, I** and **K**) Images were obtained by merging the FITC (green) and DAPI signals (blue). (**B**, **D**, **F**, **H**, **J** and **L**) Images were obtained by merging the fluorescence (DAPI signals) with the DIC images.

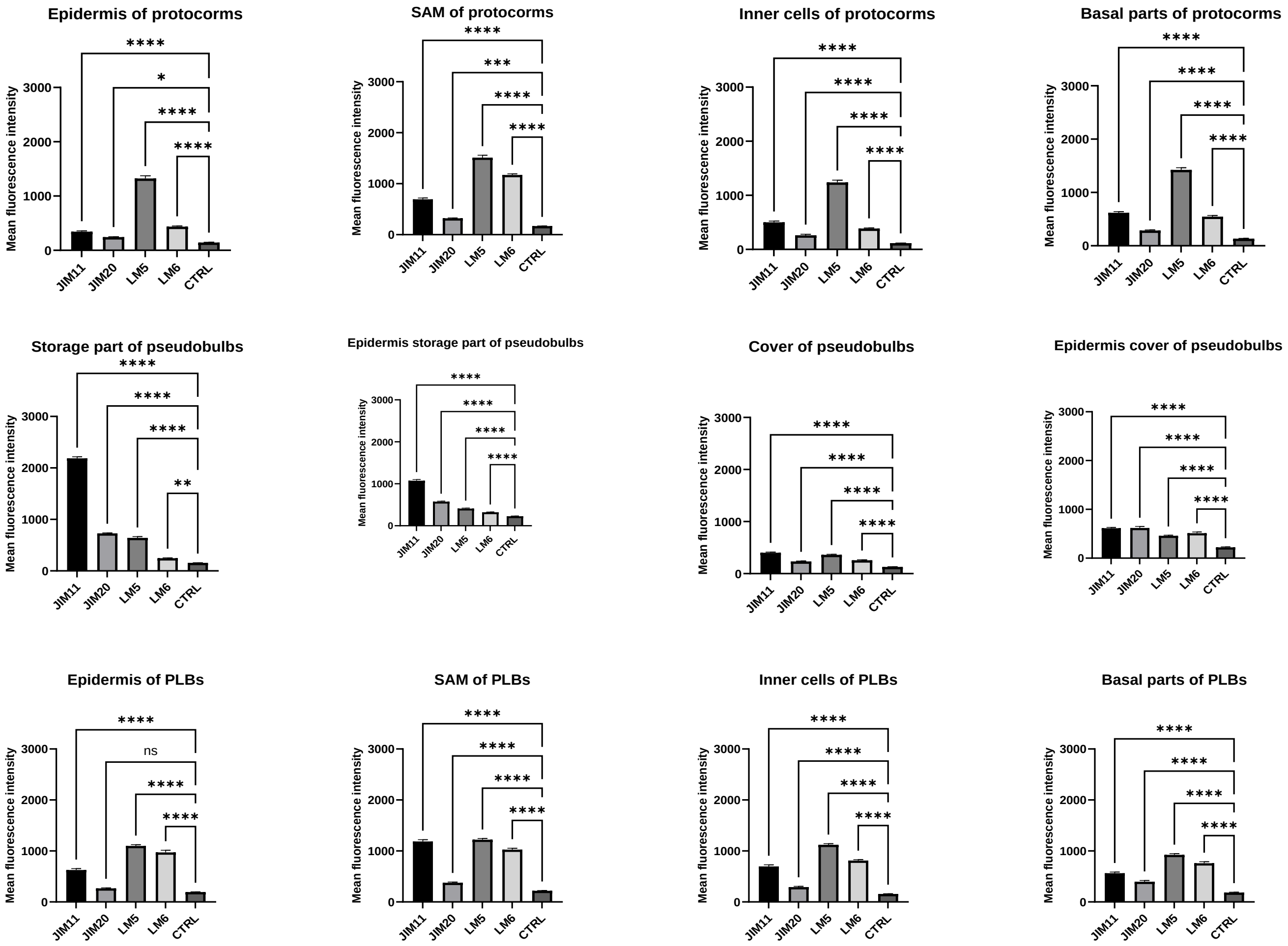

Figure S 2 Comparison of the mean fluorescence intensity of the FITC antibody conjugated to the JIM11, JIM20, LM5 and LM6 antibodies and cell wall autofluorescence (CTRL) in the cells of the analysed structures. The values are the means ± SE (one-way ANOVA). The statistical significance of the differences between the means is shown in the figures above as follows: (ns) P > 0.05,(*) P ≤ 0.05, (**) P ≤ 0.01, (***) P ≤ 0.001, (****) P ≤ 0.0001.

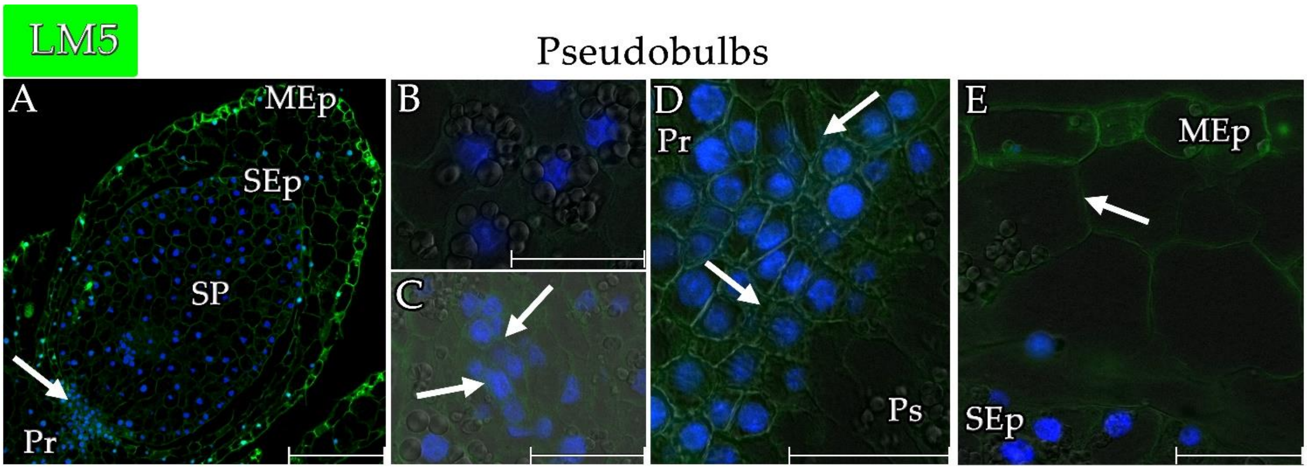

Figure S 3. Distribution of the pectin epitopes that were detected by the LM5 antibody in the analysed structures of *Liparis loeselii*. (**A**) The pseudobulbs had weak signals in most of the cell walls of pseudobulb cells. The strongest signal came from the mantle epidermis lining the storage part of the pseudobulbs (MEp). In the other parts, the signal was balanced, including in the cells of the storage part (SP), its epidermis (SEp) and the site of contact (arrow) with the protocorm (PT). (**B**) Cells with a high content of starch grains had signals in the cell walls. (**C**) Meristematic-like cells (arrows) within the storage part of the pseudobulb with signals in the cell walls. (**D**) Site of connection between a pseudobulb (Ps) and protocorm (Pr) with a weaker signal in the cell wall. There was also a signal within the xylem vessel walls (arrows). (**E**) It covered parts of the storage pseudobulb with the LM5 signalling being visible in the cell walls. Scale bar = (**Q**) 200µm or (**B**, **C** and **E**) 50µm. (**A**) Images were obtained by merging the FITC (green) and DAPI signals (blue). (**B**, **C**, **D** and **E**) Images were obtained by merging the fluorescence (FITC and DAPI signals) with the DIC images.

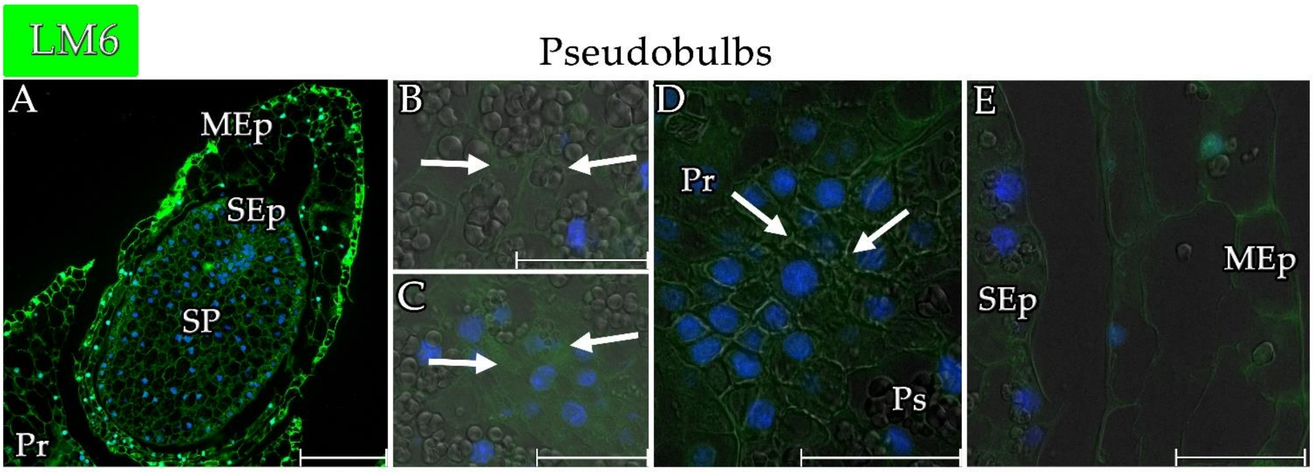

Figure S 4. Distribution of the pectin epitope that was detected by the LM6 antibody in the analysed structures of *Liparis loeselii*. (**A**) The pseudobulbs had weak signals in most of the cell walls of the pseudobulb cells. The strongest signal was visible in the mantle epidermis coat and the pseudobulb (MEp) storage part. In other parts, the signal was balanced, including in the cells of the storage part (SP), its epidermis (SEp) and the site of contact (arrow) with the protocorm (PT). (**B**) Cells with a high content of starch grains had signals in the cell walls. (**C**) Meristematic-like cells (arrows) within the storage part of the pseudobulb with signals in the cell walls. (**D**) Site of connection between a pseudobulb (Ps) and a protocorm (Pr) with signal in the walls. There were also signals within the xylem vessel walls (arrows). (**E**) It covered parts of the storage pseudobulb with LM6 signalling that was visible in the cell walls. A signal was also present in the epidermis of the coat of the pseudobulb (MEp). Scale bar = (**A**) 200µm or (**B**, **C**, **D** and **E**) 50µm. (**A**) Images were obtained by merging the FITC (green) and DAPI signals (blue). (**B**, **C**, **D** and **E**) Images were obtained by merging fluorescence (FITC and DAPI signals) with the DIC images.
